# Second-Generation Tyrosine Kinase Inhibitors (Tki) as Salvage Therapy for Resistant or Intolerant Patients to Prior TKIs

**DOI:** 10.4084/MJHID.2014.003

**Published:** 2014-01-02

**Authors:** Massimo Breccia, Giuliana Alimena

**Affiliations:** Department of Cellular Biotechnologies and Hematology, Sapienza University, Rome, Italy

## Abstract

With the advent of target therapies, imatinib became the mainstay for treatment of chronic myeloid leukemia. However, despite the brilliant results obtained with this drug, more than 30% of patients discontinue therapy in long-term due to several reasons, including failure and/or intolerance. Second-generation tyrosine kinase inhibitors (TKIs) are more potent drugs and have expanded inhibition against a broad spectrum of mutations resistant to imatinib. Both nilotinib and dasatinib have demonstrated in vitro and in vivo clinical activity against different types of mutations and various forms of resistance. However, patients with T315I mutation do not obtain an advantage from these drugs and a third generation inhibitor ponatinib, a pan-BCR drug, was tested with significant results. In this review, we report the results of second-and third-generation TKIs tested as second or third line therapy in patients resistant and/or intolerant to previous inhibitors.

## Introduction

Although the use of standard dose imatinib as first therapeutic strategy has dramatically changed the outcome of chronic myeloid leukemia patients in chronic phase, one third of patients does not achieve an optimal outcome and requires alternative therapies due to the emergence of drug resistance. Eight-year follow-up of the international IRIS study showed 85% overall survival (OS) rate, but 30% of patients had unfavourable outcome, mostly due to primary (17%) or acquired resistance (15%).^1^

In 2006, the European LeukemiaNet (ELN) group published recommendations and identified at 3, 6, 12 and 18 months different categories of patients defined as optimal or suboptimal response or failure to imatinib given as first line therapy.^2^ In 2013 the recommendations were updated^3^ and the category of suboptimal patients was excluded while resistant patients were identified also based on early evaluation of molecular response. Resistance mechanisms were studied first in vitro and then in vivo, and allowed the development of second-generation tyrosine kinase inhibitors. These agents were tested on a large series of patients resistant and/or intolerant to imatinib, providing the basis for prescription in second line.

In the present paper, we analyse the different options for patients resistant or intolerant to TKIs through a review of second-generation drugs trials performed in this category of subjects.

## Dasatinib

Dasatinib is a second-generation BCR-ABL inhibitor that has 325-fold higher potency in vivo with inhibitory activity against the majority of imatinib-resistant BCR-ABL mutants. Several studies tested the efficacy and safety of dasatinib as a second line therapy^4^. Phase II START-C trial 5 evaluated dasatinib as a single agent at the dose of 70 mg twice daily in 387 patients with resistance (75%) or intolerance (25%) to imatinib. Fifty-five percent of patients had received as prior therapy high doses of imatinib, and 10% of patients were included after failure of bone marrow transplantation. Complete hematologic remission (CHR) was achieved by 90% of patients; major cytogenetic response (MCyR) was obtained in 62% of patients with 88% of these maintaining the response at 24 months. Complete cytogenetic response (CCyR) rate was 53%, and this response was maintained in 90% of patients at 24 months. PFS at 2 years was 80%, and 2-year OS was 94%. Mutations of the kinase domain were detected at baseline in 44% of enrolled patients, with high frequency of G250E and T315I mutations. The presence of mutations at baseline, even if detected in the p-loop region, did not influence overall response rate.^5^ A subsequent sub-analysis was reported by Branford and colleagues on the development of new detectable mutations in 479 patients treated with dasatinib after imatinib failure: the results showed that the emergence of new mutations, such as T315A, F317L, V299L, occurred in only 13% of treated patients.^6^ As regards hematological adverse events of grade 3/4 reported in the first 2 years, these consisted predominantly of neutropenia (50%) and thrombocytopenia (49%). Non-hematological adverse events observed in this trial consisted prevalently of diarrhea, headache, rash and fatigue, but were of grade 3/4 in <5% of patients with a slight increase in the prevalence between the first and the second year of follow-up. Pleural effusion rate was 22%, but the majority of cases were grade 1/2 (grade 3 in less than 10%, with no grade 4) and most occurred during the first two years, of follow-up 7. Cross-intolerance between imatinib and dasatinib was not evidenced in this trial.^5^,^7^

The START-R trial included imatinib-resistant patients that were randomized, with a ratio of 2:1, to dasatinib 70 mg twice daily or high-dose imatinib (800 mg daily).^8^ After 2-year follow-up, CHR was obtained in 93% of patients treated with dasatinib and in 82% of patients treated with high-dose imatinib. A higher MCyR rate was observed among patients treated in the dasatinib arm than in the imatinib arm (53% vs. 33%; p=0.017), with a CCyR rate of 44% and 18%, respectively. MCyR was maintained in 90% of patients in the dasatinib arm and in 74% of patients in the high-dose imatinib arm. Major molecular responses (MMR) were also more frequently seen in patients treated with dasatinib than in those treated with high-dose imatinib (29% vs. 12%). As in other trials, the most frequent grade 3/4 events with dasatinib was neutropenia, thrombocytopenia and leukopenia, diarrhea, fatigue and headache.^8^ START-R study results showed that second-generation drug represents a better choice for resistant patients compared with dose escalation of imatinib.

START-A trial recruited 174 accelerated phase (AP) CML patients, with the majority of them being resistant to imatinib. At a minimum follow-up of 14 months, major hematologic response (MaHR) and CHR were achieved in 64% and 45% of patients treated with dasatinib 70 mg twice daily. MCyR and CCyR were obtained in 39% and 32% of patients, respectively. No significant differences in terms of rate of responses were observed among resistant or intolerant patients, previous stem cell transplant or presence of baseline mutations. At a median follow-up of 1 year, PFS and OS were 66% and 82%. Grade 3/4 neutropenia and thrombocytopenia occurred in 76% and 82% of patients, respectively. Diarrhoea occurred in 52% of patients with 8% being of grade 3/4, whereas pleural effusion occurred in 27% of patients (5% as grade 3/4).^9^

Dasatinib was tested also in CML blast phase (BP) and Ph+ acute lymphoblastic leukemia (ALL) (START-B and START-L trials); the former enrolled 74 myeloid blast phase (MBP) and 42 lymphoid blast phase (LBP) patients.^10^ At 8-month follow-up the rates of MaHR were 34% and 31% in MBP and LBP, respectively; MCyR rates were 31% and 50%, while CCyR rates were 27% and 43%, respectively. More resistant mutations (M244V, G250E, Y253H, E255K, E255V, T315I, F359V, H396R) were associated with lower response rates to dasatinib. Among MBP patients, the most frequently encountered AEs were diarrhoea (36%), pleural effusion (28%, 14% as grade 3/4), peripheral oedema (19%), and dyspnoea (18%). Common side effects observed in LBP patients were diarrhoea (31%), fatigue (29%), nausea and vomiting (24%). The START-L trial reported the results obtained in 36 Ph+ acute lymphoblastic leukemia (ALL).^11^ Out of 24 patients (67%) who had achieved a MaHR, 5 experienced disease progression and CCyR rate at 8-month follow-up was 58%. No differences in response rates were revealed for patients with resistant mutations compared to whole non-mutated population: T315I mutation was found at baseline in 6 patients and was associated with a worse response. The most frequently reported AEs of any grade in ALL were diarrhoea (31%), pyrexia (25%), and nausea (22%), whereas the most common grade 3/4 events, were febrile neutropenia (11%), diarrhoea (8%), and asthenia (8%). The results of trials in an advanced phase of disease showed that dasatinib is a valid option due to its large spectrum of inhibition, even if in most of the patients treated in blast crisis the responses were not long lasting.

CA180-034 study was an international, open-label, four-arm randomized phase III study, which enrolled 662 patients resistant or intolerant to prior imatinib therapy. Patients received 140 mg or 100 mg of dasatinib, both administered in one (QD) or two daily (BID) doses.^12^ Baseline features, as well as outcomes at 72 months, were similar among the different arms.^13^ CHR was achieved in 92% of the 100 mg QD arm, in 88% of the 70 mg BID arm, in 87% of the 140 mg QD arm and in 92% of the 50 mg BID arm. CCyR was achieved in 50% and 53% of the 100 mg QD and 70 mg BID arms, respectively, and in 50% and 49% of the 140 mg QD and 50 mg BID cohorts, respectively. MMR rate was 45% in the 100 mg QD cohort at the last follow-up of 72 months. OS was estimated to be 71% in the 100 mg QD arm with a cumulative incidence of death due to CML of 12.5%. PFS in the 100 mg QD arm was estimated to be 49% at 6 years. A comprehensive sub-analysis, including patients treated in phase II/III trials because resistant to imatinib, showed that the drug was active against several ABL mutations. The results showed that the drug induced high cytogenetic and molecular response rates in imatinib-resistant patients with and without mutations at baseline (including mutations at residues G250, M351, L248, Y253, E255, F359 and H396). However, mutations with half maximal inhibitory concentration (IC_50_) > 3 nM obtained a CCyR rate of 32% and MMR rate of 23% compared to CCyR rate of 53% and MMR of 38% of patients with IC_50_ < 3 nM, with also different PFS, that was 67% in IC_50_ > 3 nM group and 80% in IC_50_ < 3 nM group.^14^

A subsequent phase 3 study assessed safety and efficacy of two different schedules (140 mg QD or 70 mg BID) of dasatinib in patients in blast phase. A two-year follow-up showed that MaHRs were similar in MBP treated with the two different schedules (28%), whereas for LBP the response rate was 42% for 140 mg QD and 32% for 70 mg BID. MCyR rate was 25% for 140 mg QD and 28% for 70 mg BID in MBP patients and respective rates were 50% and 40% for LBP. Two-year OS rate with 140 mg QD and with 70 mg BID was 24% and 28% in MBP patients, respectively and 21% and 16% in LBP, respectively. In this trial a trend of better tolerability was reported among patients treated with 140 mg QD.^15^

## Nilotinib

Nilotinib (Tasigna) was rationally designed to have enhanced selectivity and potency toward BCR-ABL, with clinical activity against the majority of resistant mutations to imatinib and was recommended as second line therapy for CP and AP patients resistant or intolerant to a previous imatinib.^16^

Nilotinib was tested in a phase II trial enrolling 321 CP patients with resistance or intolerance to imatinib that received nilotinib at 400 BID. Results were reported at a median follow-up of 48 months: 94% of patients reached CHR in a median time of 1 month and 59% of patients achieved MCyR in a median time of 1.4 months, with 45% of these being CCyR; 78% of patients achieving MCyR, maintained this response. MMR rate was 28%, PFS was 57% and OS was 78%.^17^ Grade 3/4 neutropenia and thrombocytopenia occurred in 30% of patients; the most frequent non haematological side effects reported were biochemical abnormalities, including an increase of lipase/amylase, total bilirubin and hyperglycemia; other non-hematologic side effects included skin rash, nausea and headache. A mutational screening at baseline revealed that 114 out of 281 tested patients (41%) presented a mutation. The results of this sub-analysis showed that mutations with IC50 > 150 nM occurred in 14% of resistant patients and affected prevalently 3 amino acid residues (Y253H, E255K/V, F359C/V); 15% of patients had a mutation with unknown IC50. After 12 months of therapy, MCyR rates of mutated patients (both with IC50 < 150 nM and with unknown IC50) were comparable to those of non-mutated patients (MCyR 49% vs 60%, respectively). MCyR in mutated patients with IC50 > 150 nM was less favourable (19%). Disease progression rate was higher in mutated patients when compared to that of patients without mutations (46% vs 26%); in patients with IC50 > 150 nM the rate was 69%.^18^

An expanded access characterized safety of nilotinib in a large series of patients (1,422). In CP subjects, the CHR and CCyR rates were 43% and 34%, respectively. Faster responses were detected in particular in patients with sub-optimal response to imatinib, who reached 50% of CCyR. The 18-month PFS was 80%. Non-hematologic side effects reported were mild to moderate and included rash, headache, nausea and elevations in serum bilirubin and lipase that occurred in 45 and 7% of patients, respectively.^19^ In the same series of patients, 181 were in accelerated phase and 190 in blast phase (133 myeloid, 50 lymphoid and 7 unknown). As regards toxicity, myelosuppression was usually manageable with drug dose reductions or temporary interruptions, and grade 3/4 increase in serum bilirubin and lipase were infrequent.^20^

Nilotinib at the dose of 400 mg BID was tested in 105 MBP patients and 31 LBP: at 2-year follow-up, 60% of MBP and 59% of LBP had achieved a MaHR and 38% and 52% had obtained an MCyR, respectively. CCyR rate was 30% and 32%, respectively. Two-year OS was 32% for MBP and 10% for LBP patients. Fourteen patients underwent allogeneic stem cell transplant. Hematological toxicity was frequent with grade 3/4 neutropenia, thrombocytopenia and anemia occurring in 68%, 63% and 47%, respectively. Laboratory abnormalities were common, with grade 3/4 hypophosphatemia being detected in 15%, hyperbilirubinemia in 11% and lipase elevation in 11% of patients.^21^

## Bosutinib

Bosutinib (or SKI-606) is an oral once-daily dual Src/ABL inhibitor approved by FDA for the treatment of adult patients with CP, AP or BP Ph+CML, resistant or intolerant to imatinib or second-generation TKIs. It was tested in a phase I/II study, which enrolled 288 patients (200 resistant and 88 intolerant to imatinib) and the final dose of 500 mg QD was found. After a median follow-up of 24 months, 86% of patients achieved a CHR, 53% an MCyR (of them 41% had a CCyR). Among patients with CCyR, 64% of imatinib-resistant and 65% of imatinib-intolerant patients achieved MMR with CMR being obtained in 49% of imatinib-resistant and 61% of imatinib-intolerant patients. Two-year OS was 92%. Responses were seen across all types of mutants, with the exception of T315I. Most frequent side effects reported were diarrhea, rash and vomiting.^22^

Bosutinib was tested also in third line after imatinib and dasatinib and/or nilotinib failure (37 patients resistant and 50 intolerant to dasatinib; 27 patients resistant and 1 intolerant to nilotinib). The study included 118 patients that, after a median follow-up of 28 months, achieved CHR with a rate of 73% and CCyR with a rate of 24%. An MCyR was reported for 31% and 30% of dasatinib resistant and intolerant patients, with 14% and 28% achieving a CCyR, respectively. An MCyR and CCyR were observed in 35% and 27% of patients resistant to nilotinib. After 2 years, PFS was 73% with a median OS estimated to be 83%. Also in third line, bosutinib was able to overcome all types of mutations (including mutations resistant to dasatinib and nilotinib, with the most frequent being F317L, T351I, G250E and Y253H), except T315I.^23^

A recent sub-analysis of 119 patients aged over 65 years treated with bosutinib was reported in comparison with 451 younger patients.^24^ Bosutinib was administered at the dose of 500 mg/day in 3 cohorts consisting of CP patients after imatinib failure, CP patients after imatinib and dasatinib or nilotinib failure and patients in advanced phases of disease. Bosutinib was discontinued in 80% of patients aged over 65 years compared to 67% of younger patients, in 32% of cases owing to adverse events, mostly thrombocytopenia. Similar response rates were obtained in older patients in terms of CHR in the first two cohorts of patients (81% and 72%) and in terms of CCyR (38% and 23%). Rate of disease transformation was similar between older and younger patients. Two-year OS was 87% and 80% respectively in the two categories of patients, whereas 2-year PFS was 76% and 70%. Incidence of haematological side effects as well as of diarrhea was similar between older and younger patients.

Bosutinib was evaluated also in 63 patients with AP, in 48 with BP and in 23 with Ph+ALL. After a median follow-up of 8 months, 61% of AP patients and 32% of BP achieved a CHR. MCyR and CCyR rates were 48% and 33% in AP patients and 52% and 29% in BP patients, respectively. MMR and CMR rates were 15% and 4% in AP patients and 28% and 12% in BP patients, respectively. Also in advanced phase of disease, bosutinib was found to be active in mutated patients.^25^

## Ponatinib

A third generation inhibitor was recently tested in resistant/intolerant CML patients: ponatinib is a potent, synthetic, oral multi-target pan-BCR/ABL inhibitor able to block native and mutated BCR/ABL, including T315I mutation, resistant to dasatinib and nilotinib. The results of a phase 1 dose-escalation study, which enrolled 81 patients (60 with CML), showed dose-limiting side effects, which included elevated lipase and amylase and pancreatitis. Of 43 chronic phase CML patients, 72% achieved an MCyR and 44% a MMR. Similar responses were obtained in patients without mutations and with T315I.^26^ The PACE trial (Ponatinib Ph+ ALL and CML Evaluation), a phase 2 open-label trial, tested ponatinib 45 mg QD in 449 patients resistant or intolerant to dasatinib or nilotinib or with T315I mutation in different phases of the disease. Of the 444 patients included in the efficacy analysis, 267 were in CP, 83 in AP, 62 in MBP and 32 had Ph+ALL. A total of 128 patients were positive for T315I mutation. Primary endpoint was the achievement of MCyR. Overall rate of MCyR in CP patients was 54% with 44% of CCyR. Among these patients, 64 had a T315I mutation and 70% achieved an MCyR with 66% of CCyR. In AP and BP patients MaHR was evaluated as primary endpoint: overall, in AP patients the rate was 52% and of these 44% were CHR (39% in T315I mutated patients), whereas, in BP patients, the rate was 31% (29% in T315I patients). Safety evaluation revealed that the most common adverse events were hypertension, rash, abdominal pain, fatigue, headache, arterial thrombosis and hepatotoxicity. In particular 11% of patients experienced arterial thrombosis that was serious in 8% of instances. Congestive heart failure was recorded in 7% of patients, and fluid retention occurred in 23%, most commonly as peripheral edema and pleural/pericardial effusions. Other less common side effects recorded were pancreatitis (28 patients, 6%) and hemorrhagic events (24% of patients, in 5% as serious event).^27^,^28^

## Conclusions

Nilotinib and dasatinib tested as a second line after imatinib failure and/or intolerance have been proven to be effective and safe drugs, with the possibility of rescuing about 50% of patients with different forms of resistance. Bosutinib was proven effective not only in the second line, but also in patients resistant to previous therapy with imatinib and nilotinib and/or dasatinib. All these drugs are less or completely ineffective in patients carrying the T315I mutation. In this latter subset a third-generation inhibitor, ponatinib, was tested with brilliant results, and recently approved for patients with resistance to previous TKIs lines. This agent allowed the rescue of the majority of patients with T315I mutation with most of responses being maintained. In the last edition of 2013 ELN recommendations, allogeneic bone marrow transplant should be considered at the time of starting second line after failure of any type of TKIs, whereas it is recommended for patients after failure of two previous TKIs.

## Figures and Tables

**Table 1:** Summary of responses in chronic phase CML patients treated with second or third generation TKI.

TKI	Studies	No. patients/treatment	CHR	CCyR	MMR	OS	PFS

	START-C^*^	387 (dasatinib 70 mg BID)	90%	53%	-	94%	80%
	
	START-R**	101 (dasatinib 70 mg BID)	93%	44%	29%	nr	86%
	
Dasatinib	START-R	49 (high-dose imatinib 800 mg)	82%	18%	12%	nr	65%
	
	CA180-034***	167 (dasatinib 100 mg QD)	92%	50%	42%***	71%***	49%***
	
		168 (dasatinib 70 mg BID)	88%	53%	43%	70%***	47%***
	
		167 (dasatinib 140 mg QD)	87%	50%	42%	77%***	40%***
	
		168 (dasatinib 50 MG BID)	92%	49%	41%	74%***	51%***
	
Nilotinib	0321 ****	321 (nilotinib 400 mg BID)	94%	45%	28%	78%	57%
	
Bosutinib	Phase I/II**	288 (bosutinib 500 mg QD)	86%	41%	64%	92%	nr
	
Ponatinib	PACE	267 (ponatinib 45 mg QD)	nr	46%	34%	94%	80%

QD= once daily; BID= twice daily, at 2-year follow-up, CHR= complete hematological remission,

** at 2-year follow-up, CCR= complete cytogenetic remission,

*** at 6-year follow-up, MMR= major molecular response,

**** at 4-year follow-up, OS= overall survival, PFS= progression-free survival.

**Table 2:** Targets inhibited by imatinib, nilotinib, dasatinib, bosutinib, ponatinib

IMATINIB	NILOTINIB	DASATINIB	BOSUTINIB	PONATINIB
ABL	ABL	ABL	ABL	ABL
ARG	ARG	ARG	BCR-ABL	BCR-ABL
BCR-ABL	BCR-ABL	BCR-ABL	ALK	c-KIT
c-KIT	c-KIT	c-KIT	CSK	FLT3
PDGFR	PDGFR	PDGFR	FGR	FGFR
DDR1	DDR1	SRC	LYN	RET
NQO2	NQO2	YES	PKA	PDGFR
		FYN	CK1	VEGFR
		LYN	CK2	SRC
		HCK	SRC	
		LCK	RET	
		FGR	SYK	
		BLK		
		FRK		
		CSK		
		BTK		
		TEC		
		BMX		
		TXK		
		DDR1		
		DDR2		
		ACK		
		BRAF		
		EGFR		
		EPHA		
		MAPK		
		RAF		
		SLK		
		ZAK		

**Table 3:** In vitro sensitivity of more common BCR/ABL1 kinase domain mutations.

Kinase mutation	Imatinib IC50, range (nM)	Nilotinib IC50, range (nM)	Dasatinib IC50, range (nM)	Bosutinib IC50, range (nM)	Ponatinib IC50, range (nM)
**Unmutated**	260–678	<10–25	0,8-1,8	41,6	0,5
**M244V**	1,600–3,100	38–39	1,3	147,4	2,2
**L248V**	1,866–10,000	49,5–919	9,4	NA	NA
**G250E**	1,350–>20,000	48–219	1,8-8,1	179,2	4,1
**Q252H**	734–3,120	16–70	3,4–5,6	33,7	2,2
**Y253F**	>6,400–8,953	182–725	6,3–11	40	2,8
**Y253H**	>6,400–17,700	450–1,300	1,3–10	NA	6,2
**E255K**	3,174–12,100	118–566	5,6–13	394	14
**E255V**	6,111–8,953	430–725	6,3–11	230,1	36
**D276G**	1,147	35,3	2,6	25	NA
**E279K**	1,872	36,5–75	3	39,7	NA
**V299L**	540–814	23,7	15,8–18	1,086	NA
**F311L**	480–1,300	23	1,3	NA	NA
**T315I**	>6,400–>20,000	697–>10,000	137–>1,000	1,890	11
**T315A**	125	NA	760	NA	1,6
**F317L**	810–7,500	39,2–91	7,4–18	100,7	1,1
**F317V**	500	350	NA	NA	10
**M351T**	880–4,900	7,8–38	1,1-1,6	29,1	1,5
**F359V**	1,400–1,825	91–175	2,2-2,7	38,6	10
**V379I**	1,000–1,630	51	0,8	NA	NA
**L384M**	674–2,800	39–41,2	4	19,5	NA
**L387M**	1,000–1,100	49	2	NA	NA
**H396R**	1,750–5,400	41–55	1,3-3	33,7	NA
**H396P**	850–4,300	41–43	0,6-2	18,1	1,1
**F486S**	2,728–9,100	32,8–87	5,6	96,1	NA
